# AnxA5 reduces plaque inflammation of advanced atherosclerotic lesions in apoE^−/−^ mice

**DOI:** 10.1111/jcmm.12374

**Published:** 2014-09-12

**Authors:** Mathias Burgmaier, Kristof Schutters, Brecht Willems, Emiel PC van der Vorst, Dennis Kusters, Martijn Chatrou, Lucy Norling, Erik AL Biessen, Jack Cleutjens, Mauro Perretti, Leon J Schurgers, Chris PM Reutelingsperger

**Affiliations:** a Department of Internal Medicine I, University Hospital of the RWTH AachenAachen, Germany; b Department of Biochemistry, Maastricht University, Cardiovascular Research Institute MaastrichtMaastricht, The Netherlands; c VitaK BV, Maastricht UniversityMaastricht, The Netherlands; d Department of Pathology, Maastricht University, Cardiovascular Research Institute MaastrichtMaastricht, The Netherlands; e The William Harvey Research InstituteLondon, UK

**Keywords:** atherosclerosis, annexin A5, plaque inflammation, phosphatidylserine, apoptosis

## Abstract

Annexin A5 (AnxA5) exerts anti-inflammatory, anticoagulant and anti-apoptotic effects through binding cell surface expressed phosphatidylserine. The actions of AnxA5 on atherosclerosis are incompletely understood. We investigated effects of exogenous AnxA5 on plaque morphology and phenotype of advanced atherosclerotic lesions in apoE^−/−^ mice. Advanced atherosclerotic lesions were induced in 12 weeks old Western type diet fed apoE^−/−^ mice using a collar placement around the carotid artery. After 5 weeks mice were injected either with AnxA5 (*n* = 8) or vehicle for another 4 weeks. AnxA5 reduced plaque macrophage content both in the intima (59% reduction, *P* < 0.05) and media (73% reduction, *P* < 0.01) of advanced atherosclerotic lesions of the carotid artery. These findings corroborated with advanced lesions of the aortic arch, where a 67% reduction in plaque macrophage content was observed with AnxA5 compared to controls (*P* < 0.01). AnxA5 did not change lesion extension, plaque apoptosis, collagen content, smooth muscle cell content or acellular plaque composition after 4 weeks of treatment as determined by immunohistochemistry in advanced carotid lesions. *In vitro,* AnxA5 exhibited anti-inflammatory effects in macrophages and a flow chamber based assay demonstrated that AnxA5 significantly inhibited capture, rolling, adhesion as well as transmigration of peripheral blood mononuclear cells on a TNF-α-activated endothelial cell layer. In conclusion, short-term treatment with AnxA5 reduces plaque inflammation of advanced lesions in apoE^−/−^ mice likely through interfering with recruitment and activation of monocytes to the inflamed lesion site. Suppressing chronic inflammation by targeting exposed phosphatidylserine may become a viable strategy to treat patients suffering from advanced atherosclerosis.

## Introduction

Atherogenesis starts with endothelial dysfunction and transmigration of circulating inflammatory cells into the vessel wall 1. Vascular inflammation not only causes atherosclerotic disease progression but is also a major contributor to plaque vulnerability 2,3. Therefore, the modulation of vascular inflammation could be an interesting treatment option for patients with advanced atherosclerosis to prevent disease progression as well as to reduce plaque vulnerability with consecutive cardiovascular events 4.

Statins have been demonstrated to reduce plaque inflammation and vascular apoptosis in addition to their effects on cholesterol metabolism 5–7 and are currently recommended for primary as well as secondary prevention after acute myocardial infarction. However, statins are not tolerated well in subgroups of patients and there is a current lack of medications available which target vascular inflammation and reduce both progression of atherosclerosis and plaque vulnerability.

Annexin A5 (AnxA5) is a single chain protein that belongs to the annexin gene superfamily and is known for its binding to phosphatidylserine (PS) with high affinity in a calcium-dependent manner 8. PS is expressed on stressed and dying cells, and AnxA5 has been demonstrated to exert anti-inflammatory, anti-apoptotic and anticoagulant properties *via* binding to PS and shielding the stressed or dying cells from inflammatory cell contact 9,10. Previous studies demonstrated beneficial effects of AnxA5 on restenosis and vein graft patency in murine models 11,12.

Taken together, treatment with AnxA5 might be a promising option to modulate vascular inflammation to reduce atherosclerotic lesion progression and plaque vulnerability in patients with advanced atherosclerosis. Thus, we investigated the effect of exogenous AnxA5 on plaque morphology and phenotype of advanced lesions in a murine model of atherosclerosis.

## Methods

### Expression and purification of AnxA5

AnxA5 was prepared as described previously 13. Briefly, AnxA5 was expressed in *Escherichia coli* M15 (*pREP4*) (Qiagen, Venlo, the Netherlands), which were transformed with pQE30Xa (Qiagen) containing cDNA of human AnxA5. Bacteria were harvested and lysed by sonification. Cell debris was removed by centrifugation. His-tagged anxA5 was isolated from supernatant by chromatography using nickel columns (GE Healthcare, Eindhoven, the Netherlands) and an imidazole gradient. Purified proteins were checked on homogeneity (MALDI-TOF/TOF) and PS binding activity (ellipsometry) as described elsewhere 13. All AnxA5 samples contained less than one endotoxin unit per ml as determined by Endosafe PTS spectrophotometer (Charles River, Leiden, the Netherlands).

### ApoE^−/−^-mouse model of atherosclerosis

Advanced atherosclerotic lesions were induced in 12 weeks-old male apoE^−/−^ mice using both a Western type diet (0.25% cholesterol) (Abdiets, Woerden, the Netherlands) and a collar with an inner diameter of 0.3 mm around the carotid artery as described elsewhere 14. 5 weeks after collar placement mice were randomly divided into two groups receiving either AnxA5 (1 mg/kg AnxA5) or vehicle i.p. three times a week. The dosage and route of application used in our mouse experiment is similar to what has been described to be effective in murine models by other groups 11,12 and is based on a blood clearance study in which we determined the half-life of AnxA5 following i.p. injection of radiolabelled AnxA5 to be 5.64 ± 2.33 hrs. Mice were killed after another 4 weeks of Western type diet feeding and the right carotid artery and the aortic arch were excised for immunohistochemical analysis. Blood was collected for analysis of leucocyte subsets using flow cytometry.

### Histology and immunohistochemistry

Paraffin sections were stained with haematoxylin/eosin (Klinipath, Duiven, the Netherlands) to determine the volume of atherosclerotic lesions. Quantitative analysis of lesions was performed with Qwin histomorphometry software on seven sections (60 μm apart) of each mouse. Parallel sections were stained with monoclonal rat antimouse Mac-3 (BD Pharmingen, Breda, the Netherlands), cleaved caspase-3 (Cell Signaling Tech), TUNEL (*in situ* cell death detection kit, POD – Roche Applied Science, Woerden, the Netherlands) and αSMactin (Abcam, Cambridge, UK) to stain macrophages, apoptotic cells and smooth muscle cells, respectively. Antibodies were visualized with a Nova-RED substrate (Vector Laboratories, Inc, Burliname CA, USA). Sections were counterstained with haematoxylin and mounted with imsol (Klinipath, Duiven, the Netherlands) and entellan (Merck). To determine the acellular area, a Martius, Scarlet and Blue staining was performed. In negative controls, incubation with primary antibody was omitted. Picrosirius Red staining was performed to analyse the amount of collagen as described elsewhere 15.

### Flow cytometry

Using subset-specific antibodies we analysed T cells, B cells, NK cells, granulocytes, monocytes and relevant subsets of these populations as described elsewhere 16. All measurements were performed on a FACS Canto II (BD Biosciences, Breda, the Netherlands) and analysis of acquired data was performed with FACS Diva software (BD Biosciences).

### Flow adhesion assay

Flow adhesion assay was performed as previously described 17. Briefly, human umbilical vein endothelial cells were seeded on gelatin-coated μ-Slides VI0.4 (Ibidi, Martinsried, the Netherlands) and confluent monolayers were stimulated with TNFα (10 ng/ml; R&D Systems, Abingdon, Oxon, UK) overnight. Human peripheral blood mononuclear cells (PBMCs) were freshly isolated from healthy volunteers by double-density gradient centrifugation as described elsewhere 18,19. PBMCs were diluted to 1 × 10^6^/ml in Dulbecco's PBS supplemented with Ca^2+^ and Mg^2+^, and incubated with or without AnxA5 (20 nM) at 37°C for 10 min. before start of the flow experiment. PBMCs were perfused over endothelial monolayers at a constant shear stress of 1 dyne/cm^2^ using a syringe pump (Harvard Apparatus, Holliston MA, USA). Shown are normalized results of at least three independent experiments each consistent of *n* ≥ 5.

### Bone marrow-derived macrophage isolation and culture

Bone marrow cells were isolated from femurs and tibiae of C57Bl6 mice. Cells were cultured in RPMI-1640 (Life Technologies, Bleiswijk, the Netherlands) with 10% heat-inactivated foetal calf serum (Bodinco B.V., Alkmaar, the Netherlands), penicillin (100 U/ml), streptomycin (100 μg/ml) and L-glutamine 2 mM (all Life Technologies, Bleiswijk, the Netherlands) supplemented with 20% L929-conditioned medium (LCM) for 8-9 days to generate bone marrow-derived macrophages, as described previously 20.

Macrophages were seeded at 350,000 cells per well in 24 wells plates, incubated for 24 hrs with AnxA5, washed and stimulated with 10 ng/ml LPS for 6 hrs. IL-10 and TNF-α ELISA assays (all Invitrogen, Bleiswijk, the Netherlands) were performed on conditioned medium according to manufacturer's instructions. Analysis was performed with a micro-plate reader (Bio-Rad, Veenendaal, the Netherlands) at 450 nm.

### Statistical analysis

All statistical analysis was performed with IBM SPSS Statistics version 20. Continuous variables are presented as mean ± SEM. Differences between groups were analysed by Student's, Welch's or one-sample *t*-test with or without adjustment for multiple comparisons according to the Bonferroni method where appropriate. A *P* < 0.05 was regarded as statistically significant.

## Results

### Animal data

There was no change in bw, mortality, total cholesterol or triglyceride levels between mice injected with AnxA5 and vehicle (Table1). Furthermore, there was no change in circulating leucocyte subsets between groups (Table1).

**Table 1:** Animal data

Parameter	AnxA5 (*n* = 9)	Vehicle (*n* = 9)	*P*-value
Weight (g)	29.78 ± 0.62	28.63 ± 0.60	ns
Mortality, *n* (%)	0 (0%)	0 (0%)	ns
Total cholesterol (mmol/l)	26.40 ± 5.07	25.83 ± 2.59	ns
Triglyceride (mmol/l)	1.57 ± 0.14	1.46 ± 0.17	ns
Granulocytes (% viable cells)	29.6 ± 3.5	24.7 ± 3.2	ns
Monocytes (% viable cells)	13.3 ± 1.2	12.4 ± 1.4	ns
Ly6high (% viable cells)	7.9 ± 0.7	7.1 ± 0.9	ns
Ly6intermediate (% viable cells)	1.3 ± 0.1	1.1 ± 0.2	ns
Ly6low (% viable cells)	4.2 ± 0.6	4.2 ± 0.7	ns
B cells (% viable cells)	36.4 ± 3.2	41.6 ± 2.9	ns
T cells (% viable cells)	11.4 ± 1.2	12.4 ± 1.1	ns
CD4+ T cells (% viable cells)	6.6 ± 0.9	6.7 ± 0.5	ns
CD8+ T cells (% viable cells)	4.2 ± 0.4	5.1 ± 0.6	ns

### Atherosclerotic lesion extension

**Figure 1:**
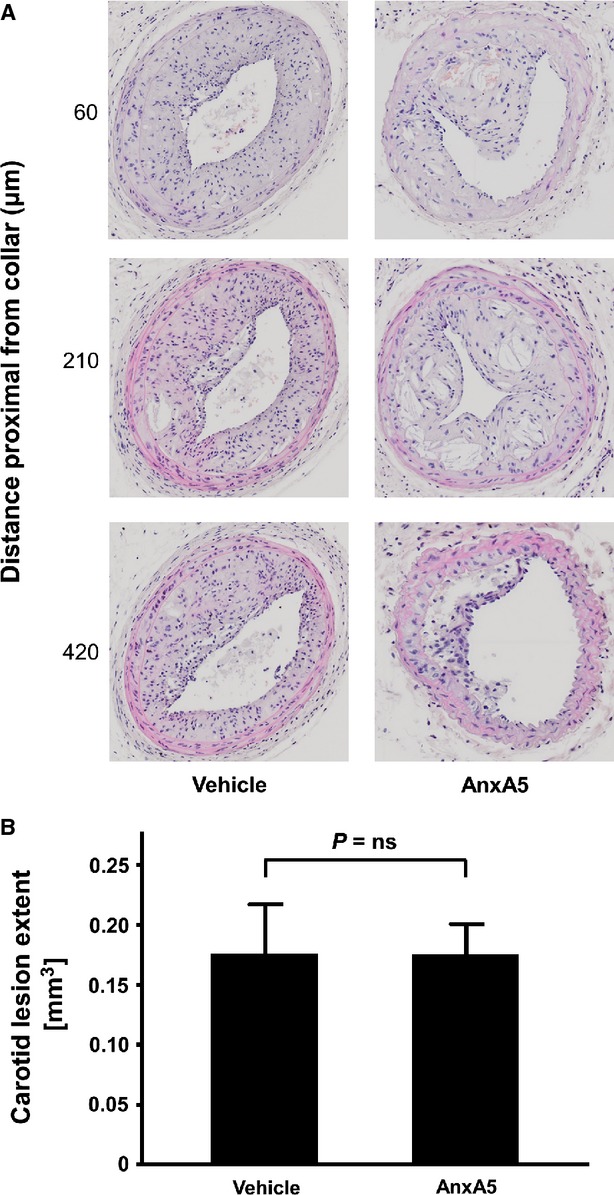
Plaque size of cross-sections of the right carotid artery obtained from vehicle and AnxA5 treated apoE^−/−^ mice is visualized using H+E stainings (**A**). (**B**) It shows statistical analysis with a bar graph; *n* = 6, *P* = ns.

To investigate the effect of AnxA5 on lesion extent of advanced atherosclerotic lesions, we quantified total plaque volume of advanced carotid lesions. As demonstrated in Figure1, there was no change in carotid artery atherosclerosis between AnxA5 (0.175 ± 0.026 mm^3^) and vehicle treated mice (0.175 ± 0.042 mm^3^, *P* = ns).

### Atherosclerotic macrophage content

As plaque inflammation is of major importance for both atherosclerotic disease progression and plaque vulnerability, we evaluated the effects of AnxA5 on plaque macrophage content using Mac-3 as a cell type-specific marker.

**Figure 2:**
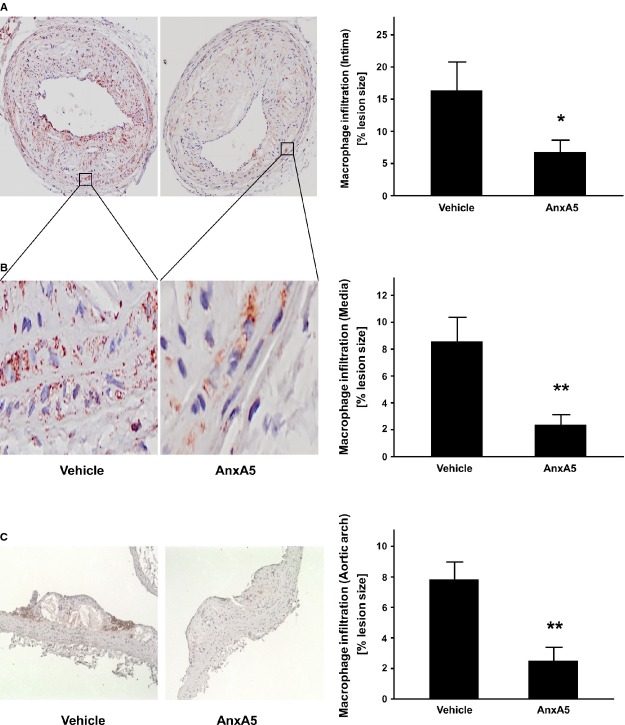
AnxA5 decreases plaque macrophage content compared to vehicle. Representative pictures from Mac-3-stained cross-sections of the carotid artery demonstrating macrophage content in the intima (**A**) and media (**B**, image in high-power view) is depicted. (**C**) It displays representative images of advanced lesions of the aortic arch. Respective bar graphs are shown (right hand side); *n* = 5–9, *P < 0.05, **P < 0.01.

Compared to control (vehicle), AnxA5 significantly reduced plaque macrophage content in the intima (59% reduction; 6.7 ± 2.0% *versus* 16.2 ± 4.6% for AnxA5 *versus* controls, *P* < 0.05, Fig.2A). A similar effect was observed in the media, where treatment with AnxA5 resulted in a 73% reduction in macrophage content (2.3 ± 0.8% *versus* 8.5 ± 1.8% for AnxA5 *versus* controls, *P* < 0.01, Fig.2B).

These findings could be confirmed in advanced lesions of the aortic arch, where a 67% reduction in plaque macrophage content was present with AnxA5 compared to controls (2.5 ± 0.9% *versus* 7.8 ± 1.2% for AnxA5 *versus* vehicle, *P* < 0.01, Fig.2C).

### Apoptosis and plaque composition

**Figure 3:**
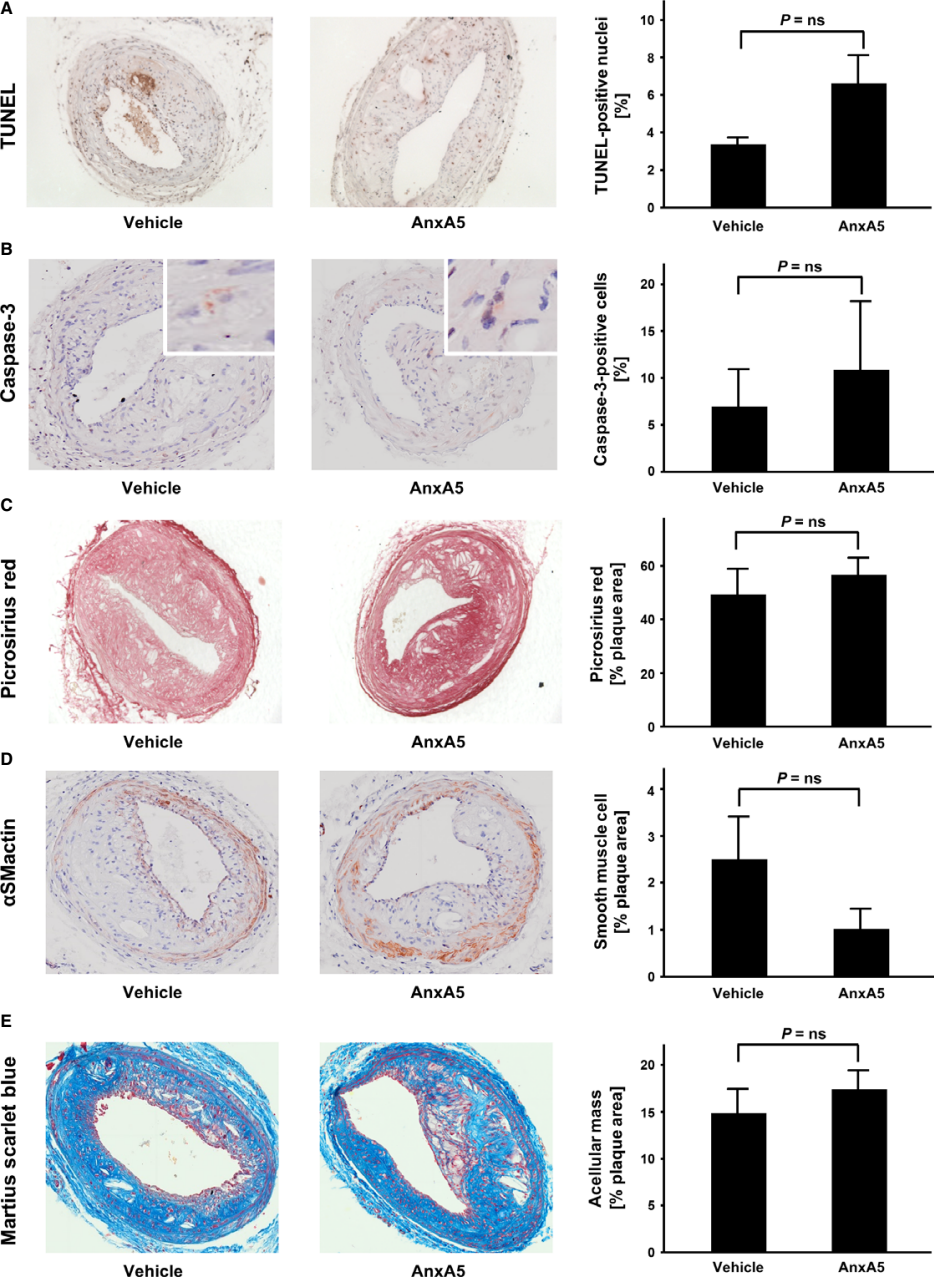
Further plaque composition of advanced carotid lesions: TUNEL-and Caspase-3 stainings (**A** and **B**) are displayed to visualize plaque apoptosis. Plaque collagen content is visualized by picrosirius red staining (**C**). Smooth muscle cell content (**D**) and acellular mass (**E**) was investigated with αSMactin– and Μartius, Scarlet and Blue staining, respectively. Representative images are shown (left hand side) and a quantification with bar graphs (right hand side) is included; *n* = 5–8; *P* = ns.

As AnxA5 is known to have anti-apoptotic effects through specific binding to PS, we performed both TUNEL and caspase-3 staining. There was no difference in apoptosis quantitatively in advanced lesions of mice injected with AnxA5 *versus* controls (Fig.3A and B).

Furthermore, there was no difference in plaque collagen content, smooth muscle cell content or acellular plaque composition after 4 weeks of treatment as determined by immunohistochemistry in advanced carotid lesions (*P* = ns, Fig.3C–E).

### Anti-inflammatory effects of AnxA5

**Figure 4:**
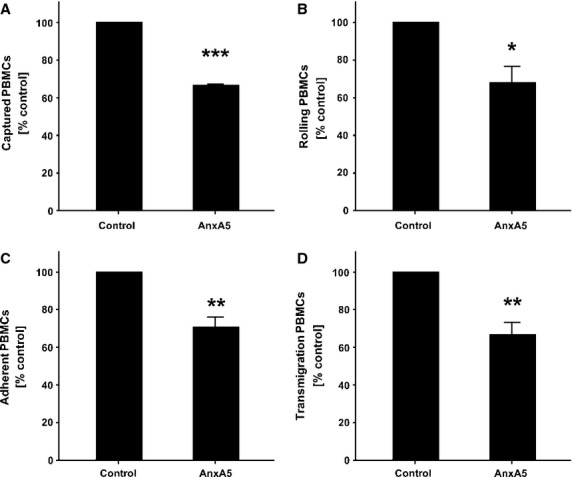
Captured (**A**), rolling (**B**), adherent (**C**) and transmigrating (**D**) PBMCs across an activated endothelial cells layer *in vitro*. Displayed are means of at least three independent experiments each consistent of *n* ≥ 5 and SEM; *P < 0.05, **P < 0.01, ***P < 0.001.

To determine if the decreased plaque inflammation observed in AnxA5 treated mice is caused by inhibition of cell transmigration, an *in vitro* flow chamber assay has been performed with isolated PBMCs and TNF-α-activated endothelial cells. As demonstrated in Figure4, AnxA5 significantly decreased capture (*P* < 0.001), adhesion (*P* = 0.005), rolling (*P* = 0.021) and transmigration (*P* = 0.002) of PBMCs. These data suggest that AnxA5 inhibits the recruitment of PBMCs across an activated endothelial cell layer into the vessel wall.

**Figure 5:**
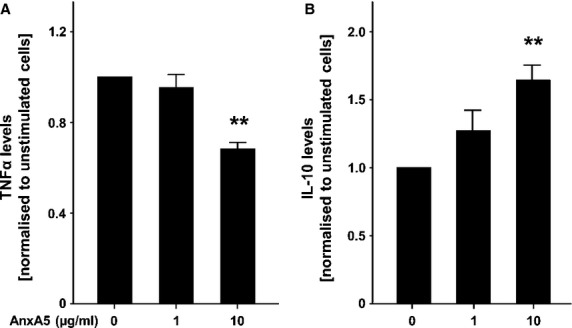
AnxA5 decreases TNF-α (**A**) and increases IL-10 (**B**) cytokine levels secreted by bone marrow-derived macrophages. Displayed are means and SEM;*n* = 4, **P ≤ 0.01.

Next, we investigated the effects of AnxA5 on TNFα and IL-10 secretion of murine bone marrow-derived macrophages. AnxA5 significantly decreased TNF-α secretion (Fig.5A), whereas the secretion of IL-10 was up-regulated with AnxA5 in a dose-dependent manner (Fig.5B), suggesting that AnxA5 induces a shift from pro-to anti-inflammatory cytokine secretion in macrophages.

## Discussion

In the present study, we demonstrate that short-term treatment with AnxA5 reduces plaque macrophage content in advanced atherosclerotic lesions in a murine model of atherosclerosis.

Coronary plaque inflammation is a major determinant of both, atherosclerotic disease progression as well as plaque vulnerability and the risk of future cardiovascular events. Apart from statins with their modest anti-inflammatory effects, there are no drugs available for patients with advanced atherosclerosis which specifically target plaque inflammation. However, these drugs may be useful to decrease both atherosclerosis progression and plaque vulnerability and thus may reduce future cardiovascular events.

AnxA5 is a 35 kD endogenous protein with an ability to bind PS with high affinity. As PS is expressed on stressed and dying cells, AnxA5 is currently a promising vector for tumour imaging and targeted drug delivery in oncology as well as for the visualization of vulnerable plaques in patients (for review see 8,9,21). In addition to these potential imaging applications of AnxA5, the protein might be used to influence plaque inflammation *via* binding to PS and thus shielding stressed or dying cells from inflammatory cell contact. Recent studies from Ewing *et al*. demonstrated that AnxA5 has a beneficial effect on restenosis and vein graft patency in murine models of atherosclerosis 11,12. In both studies the beneficial effect of AnxA5 was associated with a decrease in inflammation. However, the effect of AnxA5 on plaque inflammation is still unclear. We extend the current knowledge by demonstrating that a short-term treatment of AnxA5 inhibits plaque inflammation in a murine model of advanced atherosclerosis. Furthermore, we could demonstrate that AnxA5 reduces the transmigration of PBMCs through a TNF-α activated endothelial cell layer and induces a shift from pro-to anti-inflammatory cytokine expression in macrophages, which may be involved in the reduction in plaque inflammation observed with AnxA5 in this mouse model. As plaque inflammation is a combined feature of both atherosclerotic disease progression and plaque vulnerability, it needs to be determined in future studies if beneficial effects of AnxA5 on plaque inflammation can be translated to decreased cardiovascular events in patients suffering from advanced atherosclerosis.

Although we could demonstrate that AnxA5 reduces PBMC transmigration and induces a shift towards an anti-inflammatory phenotype *in vitro*, we cannot exclude additional mechanisms which might be involved in decreased plaque inflammation observed with AnxA5 treatment. Specifically, apoptotic cells are known to secrete lysophosphatidylcholine which acts as chemoattractant for phagocytes 22. Recently, AnxA5 has been shown to bind to lysophosphatidylcholine and thus to inhibit leucocyte chemotaxis in an peritoneal recruitment model 23. Therefore, AnxA5 might inhibit macrophage infiltration because of binding and inhibiting the chemotactic effect of lysophosphatidylcholine which is secreted from apoptotic cells within atherosclerotic lesions. Furthermore, AnxA5 is known to have anticoagulant properties and to inhibit thrombin formation catalysed by endothelial cells 24,25. Thrombin in turn is known to have pro-inflammatory and pro-atherosclerotic effects (for review, see 26). Thus, the anticoagulant effects of AnxA5 may be involved in the decrease in plaque inflammation observed with AnxA5.

In addition, AnxA5 inhibits apoptosis by binding to PS and thereby shielding the apoptotic cell from contact with phagocytes 13,27. As apoptosis is a feature of plaque vulnerability and progression, the influence of AnxA5 on plaque inflammation observed in this study may be related to the modulation of plaque apoptosis by AnxA5. However, we did not observe differences in plaque apoptosis as determined by both TUNEL and Caspase-3 stainings, suggesting that the effect of AnxA5 on apoptosis is not involved in the decrease in plaque inflammation.

In conclusion, short-term treatment of AnxA5 reduces plaque inflammation in a murine model of atherosclerosis through interfering with recruitment and activation of monocytes to the inflamed lesion site. Future studies are needed to determine if AnxA5-targeting of exposed PS is a useful strategy to suppress chronic plaque inflammation in patients with advanced atherosclerosis.
